# Optogenetic modulation of electroacupuncture analgesia in a mouse inflammatory pain model

**DOI:** 10.1038/s41598-022-12771-8

**Published:** 2022-05-31

**Authors:** I-Han Hsiao, Hsien-Yin Liao, Yi‑Wen Lin

**Affiliations:** 1 College of Chinese Medicine, Graduate Institute of Acupuncture Science, China Medical University, 91 Hsueh-Shih Road, Taichung, 40402 Taiwan; 2 Department of Neurosurgery, China Medical University Hospital, Taichung, 404332 Taiwan; 3 College of Chinese Medicine, School of Post-Baccalaureate Chinese Medicine, China Medical University, 91 Hsueh-Shih Road, Taichung, 40402 Taiwan; 4 Chinese Medicine Research Center, China Medical University, Taichung, 40402 Taiwan

**Keywords:** Neuroscience, Physiology, Neurology

## Abstract

Peripheral tissue damage and associated inflammation can trigger neuroplastic changes in somatic pain pathways, such as reduced neuronal firing thresholds and synaptic potentiation, that ultimately lead to peripheral sensitization and chronic pain. Electroacupuncture (EA) can relieve chronic inflammatory pain, but the underlying mechanisms are unknown, including the contributions of higher pain centers such as somatosensory cortex (SSC). We investigated these mechanisms using optogenetic modulation of SSC activity in a mouse inflammatory pain model. Injection of Complete Freund's Adjuvant into the hind paw reliably induced inflammation accompanied by reduced mechanical and thermal pain thresholds (hyperalgesia) within three days (mechanical: 1.54 ± 0.13 g; thermal: 3.94 ± 0.43 s). Application of EA produced significant thermal and mechanical analgesia, but these responses were reversed by optogenetic activation of SSC neurons, suggesting that EA-induced analgesia involves modulation of central pain pathways. Western blot and immunostaining revealed that EA also attenuated CaMKIIα signaling in the dorsal root ganglion, central spinal cord, SSC, and anterior cingulate cortex (ACC). In contrast, optogenetic activation of the SSC induced CaMKIIα signaling in SSC and ACC. These findings suggest that AE can relieve inflammatory pain by suppressing CaMKIIα-dependent plasticity in cortical pain pathways. The SSC and ACC CaMKIIα signaling pathways may be valuable therapeutic targets for chronic inflammatory pain treatment.

## Introduction

Chronic inflammatory pain drastically reduces patient quality of life and constitutes a major global healthcare and economic burden. Moreover, many currently available treatments have intolerable side-effects, such as gastric ulcers from prolonged use of non-steroidal anti-inflammatory drugs (NSAIDs)^1^, bowel dysfunction caused by morphine^2^, and immune system suppression produced by steroids^3^. Thus, alternative approaches are needed for the management of chronic pain, including integrative treatments with less reliance on these pharmacotherapies. The development of such treatments requires a more thorough understanding of the pathomechanims of inflammatory pain to identify new therapeutic targets.

Numerous factors modulate the sensitivity of peripheral nociceptive neurons, including prostaglandins, bradykinin, histamine, substance P, H^+^, and ATP. Pain signals converge on the cell bodies of dorsal root ganglion (DRG) neurons and are then transmitted to the spinal cord (SC) and higher brain centers. A cardinal feature of chronic inflammatory pain is hyperalgesia due to reduced firing thresholds of nociceptive neurons, termed peripheral nerve sensitization^4^. The sensitization process induced by local inflammation has been studied extensively in mice injected with pro-inflammatory agents such as capsaicin, carrageenan, collagen, or Complete Freund's Adjuvant (CFA), or subjected to mechanical tissue damage such as experimental ankle sprain. Among these inducers, CFA is widely used as it mimics inflammatory pain in humans^5^.

Elucidation of the neural circuits modulating pain, particularly at the central level, requires improved methods for experimentally manipulating the excitability of excitatory and inhibitory neurons. Such effects may be induced by direct pharmacological modulation of neuronal excitability or by altering the functions of surrounding glial cells, but it is difficult to target diffusible compounds to specific regions of interest. A promising alternative is optogenetic modulation, in which local neurons are infected with adenovirus-associated virus (AAV) encoding light-sensitive proteins called opsins^6–8^ that alter membrane ionic permeability upon laser light stimulation. For instance, channelrhodopsin is a light-sensitive cation channel that can be activated using ~ 470 nm blue wave light to initiate a depolarizing current, thereby increasing excitability and neuronal firing^9^. Alternatively, the light-activated chloride transporter halorhodopsin (activated at ~ 589 nm) and the light-activated proton efflux pump archaerhodopsin (activated at ~ 550 nm) can inhibit neuronal activity through membrane hyperpolarization^8,9^. Targeted transfection of these optogenetic proteins allows for the selective activation or inhibition of local neurons by light stimulation concomitantly with various behavioral tests to identify circuits responsible for the cortical processing and modulation of pain^10,11^.

The activation of both peripheral and central pain pathways requires the opening of calcium-permeable ion channels. The resultant calcium influx activates the calcium-sensitive kinase CaMKIIα, which is abundantly expressed at multiple levels of the pain transmission pathway, including the DRG, SC, and cortex, and is strongly implicated in pain sensation^12,13^. For instance, neuronal CaMKII signaling pathway activation has been observed during nerve injury-induced neuropathic pain at the SC level^13^. Further, chronic pain induction increases neuronal excitability in the anterior cingulate cortex (ACC) and concomitantly upregulates CaMKII expression^14^. Upon activation, CaMKIIα can further trigger a number of downstream signaling factors, such as the mitogen-activated protein kinases (MAPKs) extracellular signal-regulated protein kinases 1/2 (ERK1/2), p38, and c-Jun N-terminal kinase/stress-activated protein kinase (JNK), and various transcription factors that alter neuronal responsiveness to inflammatory pain inputs^12–14^. In addition to CaMKIIα, the PI3K-Akt-mTOR signaling axis is also involved in central sensitization^14–16^.

Acupuncture is an ancient technique of Traditional Chinese Medicine used to cure a variety of ailments, including inflammation and chronic pain. Electrical stimulation of acupuncture needles, termed electroacupuncture (EA), has been shown to increase the reproducibility and efficacy of analgesia in mouse models of inflammatory pain, neuropathic pain, and fibromyalgia^17–20^, possibly by increasing the release of endogenous opiates^21^, dopamine^22^, and adenosine^23^, and (or) by downregulating the release of pro-inflammatory interleukins, TNFα, and IFN-γ^19^. Our previous study also demonstrated that EA can reduce mechanical and thermal hyperalgesia in an inflammatory mouse model by suppressing TRPV1 signaling^19^.

In this study, we tested the hypothesis that inflammatory pain is associated with increased neural activity in the DRG, SC, somatosensory cortex (SSC), and ACC, and that EA-induced analgesia is mediated by suppression of neural activity in these regions. To this end, we established a mouse model of inflammatory pain by CFA injection and examined behavioral correlates of pain during optogenetic activation of the SSC. Consistent with our hypothesis, optogenetic activation of the SSC reversed the analgesic effect of EA. Also, pain induction enhanced CaMKII signaling in the DRG, SC, SSC, and ACC, and these responses were suppressed by EA. Moreover, optogenetic activation of the SSC reversed the local reduction in CaMKII signaling triggered by EA, suggesting that CaMKII-induced neuroplastic processes in the SSC and ACC are critical for central pain sensitization. Our results therefore indicate that the analgesic effect of EA is dependent on CaMKII signaling in higher cortical pathways. We also provide evidence that inflammatory mediators modulate the CaMKII signaling pathway, thereby suggesting new potential therapeutic targets for treating inflammatory pain.

## Results

### CFA-induced inflammatory pain sensitization and modulation of EA analgesia by optogenetic stimulation of the SSC

**Figure 1:**
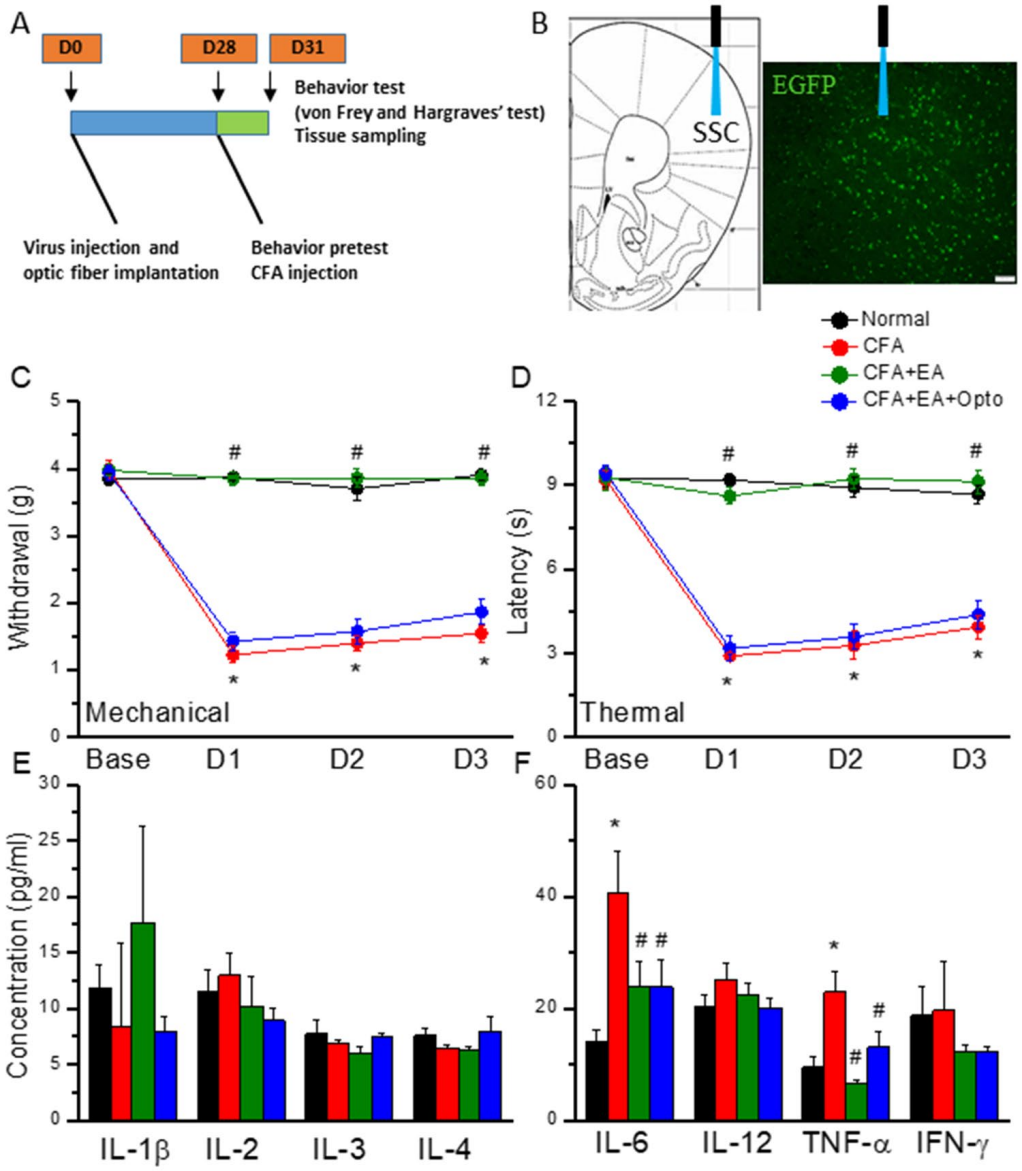
Illustrate the experimental protocol, mechanical, thermal hyperalgesia, and inflammatory mediators in 4 groups. (**A**, **B**) Illustration of the experimental protocol. (**C**) Mechanical threshold from the von Frey tests. (**D**) Thermal latency from the Hargreaves’ test. (**E**) IL-1β, IL-2, IL-3, and IL-4 (**F**) IL-6, IL-12, TNF-α, and IFN-γ. *Indicates statistical significance when compared to the normal mice. ^#^Indicates statistical significance when compared to the CFA groups. n = 10 in all groups.

Mice were first injected with AAV into the SSC for subsequent optogenetic modulation. Inflammatory pain sensitization (hyperalgesia) was induced in these mice by CFA injection into the hind paw and confirmed by measuring the paw withdrawal thresholds to mechanical stimulation by von Frey filament test. The experimental design is illustrated in Fig. 1A,B. Mice receiving intraplantar CFA injection demonstrated significantly lower paw withdrawal thresholds than control mice (1.54 ± 0.13 g vs. 3.91 ± 0.07 g, *p < 0.05, n = 9 mice per group) (Fig. 1C, responses of CFA-treated mice indicated by red circles and control responses by black circles). The paw withdrawal threshold was significantly increased in CFA-treated mice by EA at the ST36 acupoint, indicating attenuation of mechanical hyperalgesia (Fig. 1C, green circles, D3 = 3.86 ± 0.11 g, ^#^p < 0.05 vs. CFA alone, while optogenetic activation of SSC layer 2/3 reversed this EA analgesia (Fig. 1C, blue circle, D3 = 1.86 ± 0.18 g, ^#^p < 0.05 vs. EA), suggesting that EA mitigates central sensitization.

We then examined if these effects extended to thermal hyperalgesia using Hargraves’ test. Indeed, intraplantar CFA injection significantly reduced the paw withdrawal latency compared to control mice (3.94 ± 0.43 s, *p < 0.05, n = 9 mice per group), indicating successful thermal pain sensitization, while EA-induced a significant analgesic effect as evidenced by longer withdrawal latency (Fig. 1D, green circle, D3: 9.14 ± 0.43 s, ^#^p < 0.05 vs. CFA). Furthermore, optogenetic manipulation successfully reversed EA analgesia, consistent with mechanical pain measurements and further supporting the SSC as a mediator of central sensitization.

### CFA enhanced and EA suppressed plasma pro-inflammatory cytokine levels

To examine whether EA also reduces peripheral inflammatory signaling, we measured plasma concentrations of inflammatory cytokines and chemokines by multiplex ELISA. While plasma IL-1β, IL-2, IL-3, IL-4, IL-12, and IFN-γ did not differ among treatment groups (Fig. 1E, p > 0.05, n = 6 mice per group), plasma IL‑6 and TNF-α concentrations were significantly elevated in CFA-injected mice compared to control mice (Fig. 1F, red column, *p < 0.05). Furthermore, EA treatment for three days significantly reduced circulating IL‑6 and TNF-α levels in model mice (Fig. 1F, green column, ^#^p < 0.05), and this effect was not altered by optogenetic stimulation (Fig. 1F, blue column, ^#^p < 0.05 vs. control and p > 0.05 vs. EA).

### CFA enhanced and EA suppressed CaMKIIα signaling pathways in mouse DRG

**Figure 2:**
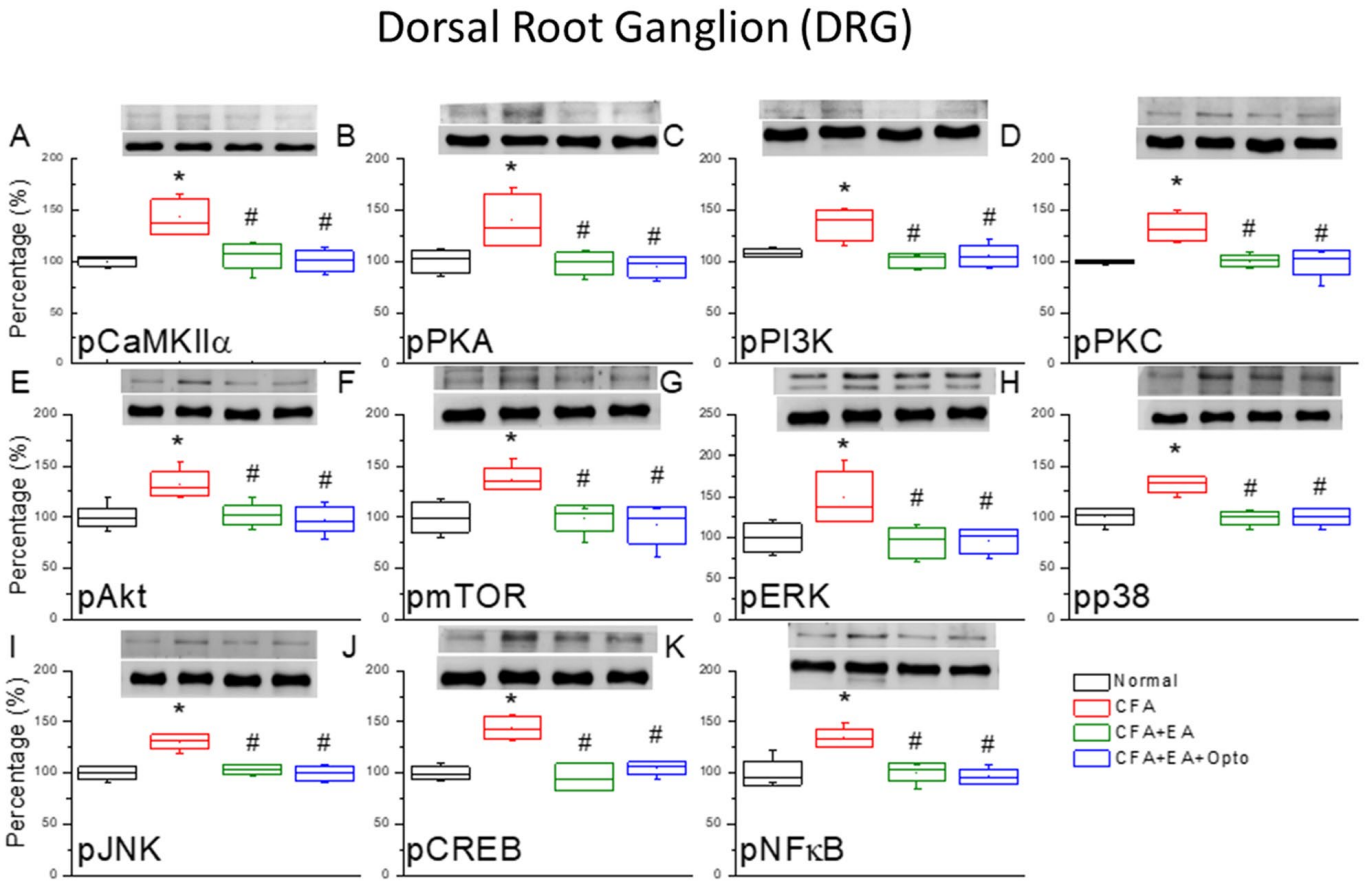
The levels of pCaMKIIα and related molecules in the mice DRG. The western blot bands have four lanes of protein expression in order of Normal, CFA, CFA + EA, and CFA + EA + Opto groups. There are significant increases of (**A**) pCaMKIIα, (**B**) pPKA, (**C**) pPI3K, (**D**) pPKC, (**E**) pAkt, (**F**) pmTOR, (**G**) pERK, (**H**) pp38, (**I**) pJNK, (**J**) pCREB, and (**K**), pNFκB protein levels in CFA group. *Indicates statistical significance when compared with the normal group. ^#^Indicates statistical significance when compared to the CFA group. n = 6 in all groups. The full Western blot bands are supplied at the end of the supplementary file.

Peripheral inflammatory pain is associated with hyperactivation of DRG neurons. Further, DRG neurons express CaMKIIα, which is implicated in the neuroplasticity underlying pain sensitization. To examine the contribution of CaMKIIα signaling in DRG neurons to CFA-induced hyperalgesia and EA analgesia, we measured the levels of phosphorylated CaMKIIα by Western blotting. Injection of CFA significantly upregulated pCaMKIIα expression (Fig. 2A, red box, 143.25% ± 7.01% of control, *p < 0.05, n = 6 mice per group) and this response by significantly attenuated by EA treatment for three days (Fig. 2A, green box, 105.89% ± 4.82% of control, ^#^p < 0.05 vs. CFA alone). Additionally, optogenetic activation after EA treatment further reduced pCaMKIIα activity in the DRG (Fig. 2A, blue box, 99.86% ± 4.25%, ^#^p < 0.05 vs. CFA alone).

We also examined the DRG expression levels of phosphorylated PKA, PI3K, and PKC, kinases activated in association with CaMKIIα. Model mice exhibited significantly higher expression levels of pPKA, pPI3K, and pPKC than control mice (Fig. 2B–D, red box, *p < 0.05, n = 6 mice per group), and this upregulation was significantly attenuated by EA (Fig. 2B–D, green box, ^#^p < 0.05 vs, CFA alone), while central optogenetic activation did not alter the effects of EA (Fig. 2B–D, blue box, ^#^p > 0.05). In addition, model mice exhibited elevated expression levels of pAkt and pmTOR (Fig. 2E,F, red box, *p < 0.05) than both EA and optogenetic groups (Fig. 2E,F, green and blue boxes, ^#^p < 0.05). Likewise, expression levels of phospho-activated MAPKs pERK1/2, pp38, and pJNK were elevated in the DRG of model mice compared to controls (Fig. 2G–I, red box, *p < 0.05) and these levels were significantly reduced by EA and optogenetic stimulation (Fig. 2G–I, green and blue boxes, ^#^p < 0.05).

These kinase pathways ultimately converge on transcription factors such as CREB and NFκB that alter gene expression patterns, so we also measured changes in activated (p)CREB and pNFκB in mouse DRG. Expression levels of both pCREB and pNFκB were increased in model mouse DRG compared to controls (Fig. 2J,K, red box, *p < 0.05, n = 6 mice per group). Expression levels were also elevated in EA and optogenetic groups (Fig. 2J,K, green and blue boxes, ^#^p < 0.05 vs. controls).

**Figure 3:**
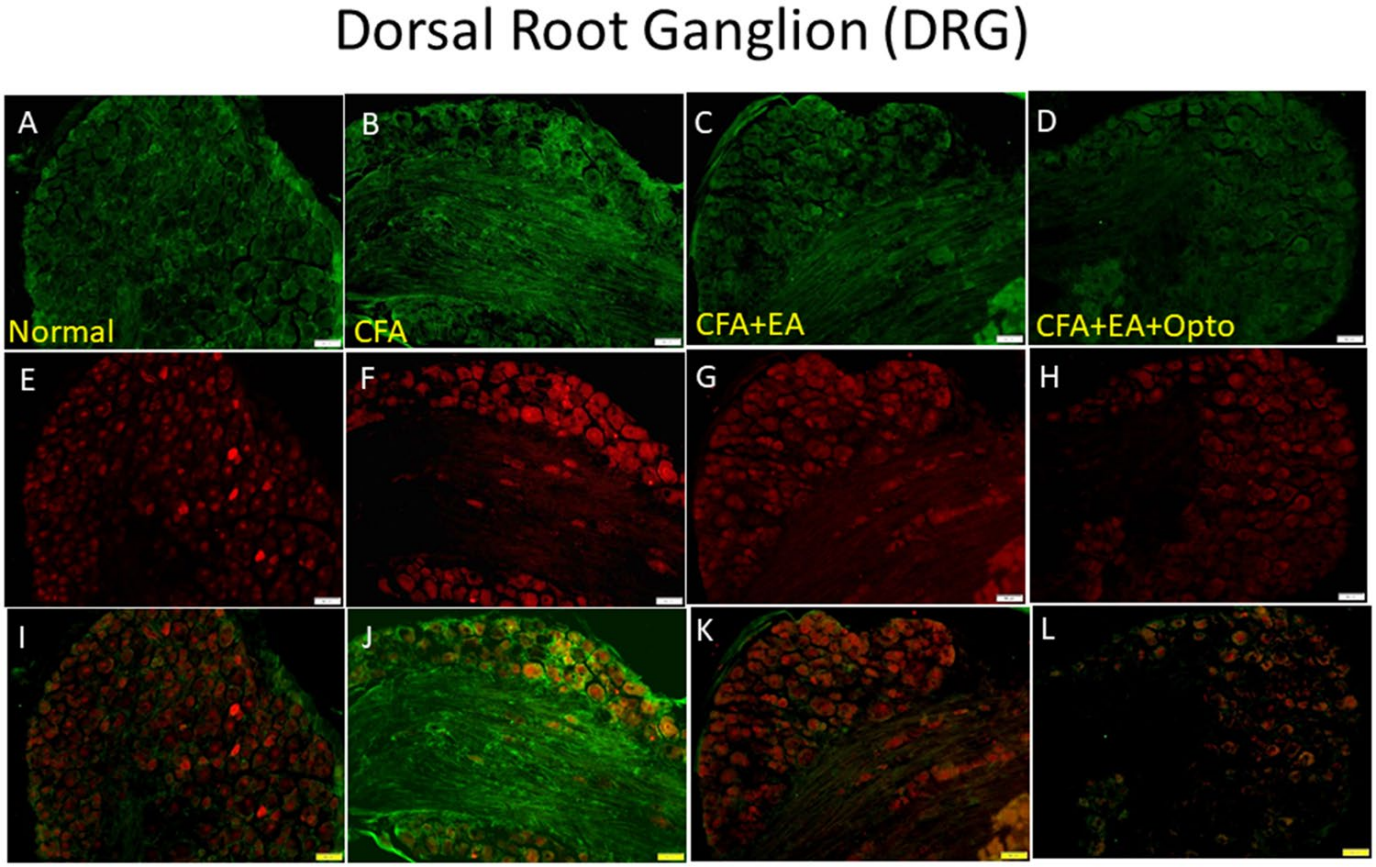
Immunofluorescence staining of pCaMKIIα, pERK, and double staining protein expression in the mice DRG. (**A**–**D**) pCaMKIIα, (**E**–**H**) pERK, and (**I**–**L**) pCaMKIIα/ pERK double staining, immuno-positive (green, red or yellow) signals in the mice DRG region. Scale bar means 100 μm. n = 4 in all groups.

Subsequently, we examined the distribution and colocalization of pCaMKIIα and pERK by immunostaining. Immunoexpression of pCaMKIIα in the mouse DRG (Fig. 3A, green fluorescence, n = 3 mice per group) was markedly enhanced by CFA injection (Fig. 3B). As expected from Western blotting results, this increase was attenuated by EA (Fig. 3C) as well as by optogenetic stimulation of the SSC (Fig. 3D). Similarly, immunoexpression of pERK in the DRG of control mice (Fig. 3E, normal group, red signal fluorescence) was upregulated by CFA injection (Fig. 3F), and this latter response was reversed by EA and optogenetic stimulation (Fig. 3G,H, respectively). Further, dual imaging revealed that pCaMKIIα and pERK were co-localized (yellow signals in merged images), and that this dual upregulation was attenuated by EA and optogenetic stimulation (Fig. 3I–L).

### EA and optogenetic stimulation attenuated CFA-induced signaling pathways in the spinal cord

**Figure 4:**
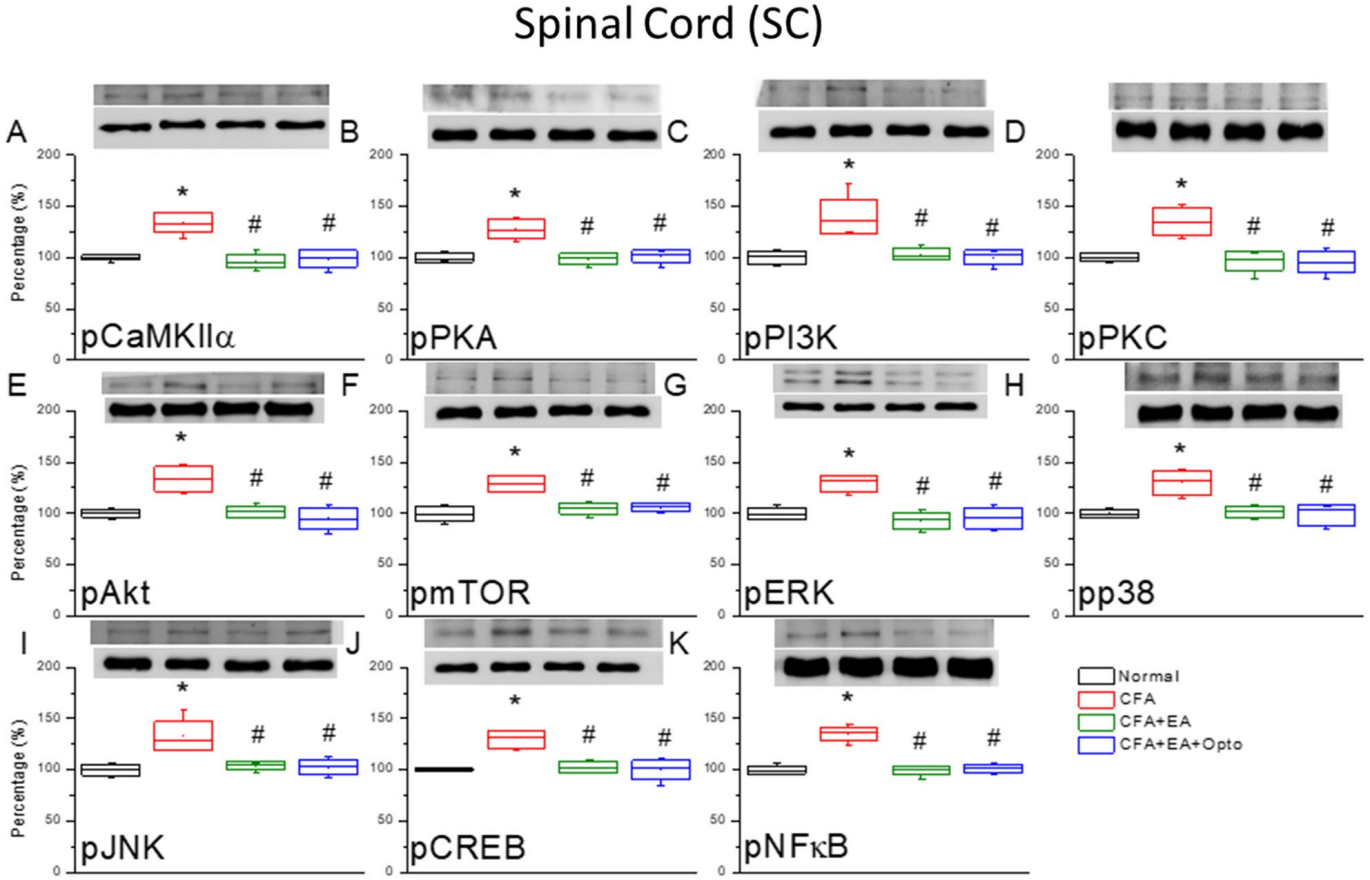
The levels of pCaMKIIα and related molecules in the mice SC. The western blot bands have four lanes of protein expression in order of Normal, CFA, CFA + EA, and CFA + EA + Opto groups. There are significant increases of (**A**) pCaMKIIα, (**B**) pPKA, (**C**) pPI3K, (**D**) pPKC, (**E**) pAkt, (**F**) pmTOR, (**G**) pERK, (**H**) pp38, (**I**) pJNK, (**J**) pCREB, and (**K**), pNFκB protein levels in CFA group. *Indicates statistical significance when compared with the normal group. ^#^Indicates statistical significance when compared to the CFA group. n = 6 in all groups. The full Western blot bands are supplied at the end of the supplementary file.

We then examined if EA also suppresses CFA-induced inflammatory pain by modulating these signaling pathways in SC. Three days after inflammatory pain induction, pCaMKIIα expression was markedly enhanced in the SC (Fig. 4A, red column, *p < 0.05 vs. controls, n = 6 mice per group), and this response was significantly attenuated by EA (Fig. 4A, green column, ^#^p < 0.05 vs. CFA alone) while optogenetic activation did not alter the effect of EA (Fig. 4A, blue column, ^#^p < 0.05 vs. controls and p > 0.05 vs. EA). Injection of CFA also increased the expression levels of pPKA, pPI3K, and pPKC in the SC (Fig. 4B–D, red columns, *p < 0.05 vs. controls), and these responses were reversed by both EA and optogenetic stimulation (Fig. 4B–D, green and blue columns, ^#^p < 0.05 vs. CFA alone). Model induction by CFA injection also significantly upregulated the expression levels of pAkt-pmTOR axis molecules in the SC (Fig. 4E,F, red column, *p < 0.05 vs. controls), and again these responses were partially reversed by EA and optogenetic stimulation (Fig. 4E,F, green and blue columns, ^#^p < 0.05 vs. CFA alone). In addition, CFA enhanced expression of pERK, pp38, and pJNK (Fig. 4G–I, red columns, *p < 0.05, n = 6), and these responses were similarly reversed by EA and optogenetic stimulation (Fig. 4G–I, ^#^p < 0.05, n = 6). Finally, pCREB and pNFkB exhibited qualitatively similar changes in response to CFA alone, EA, and optogenetic stimulation (Fig. 4J,K, *p < 0.05, n = 6).

**Figure 5:**
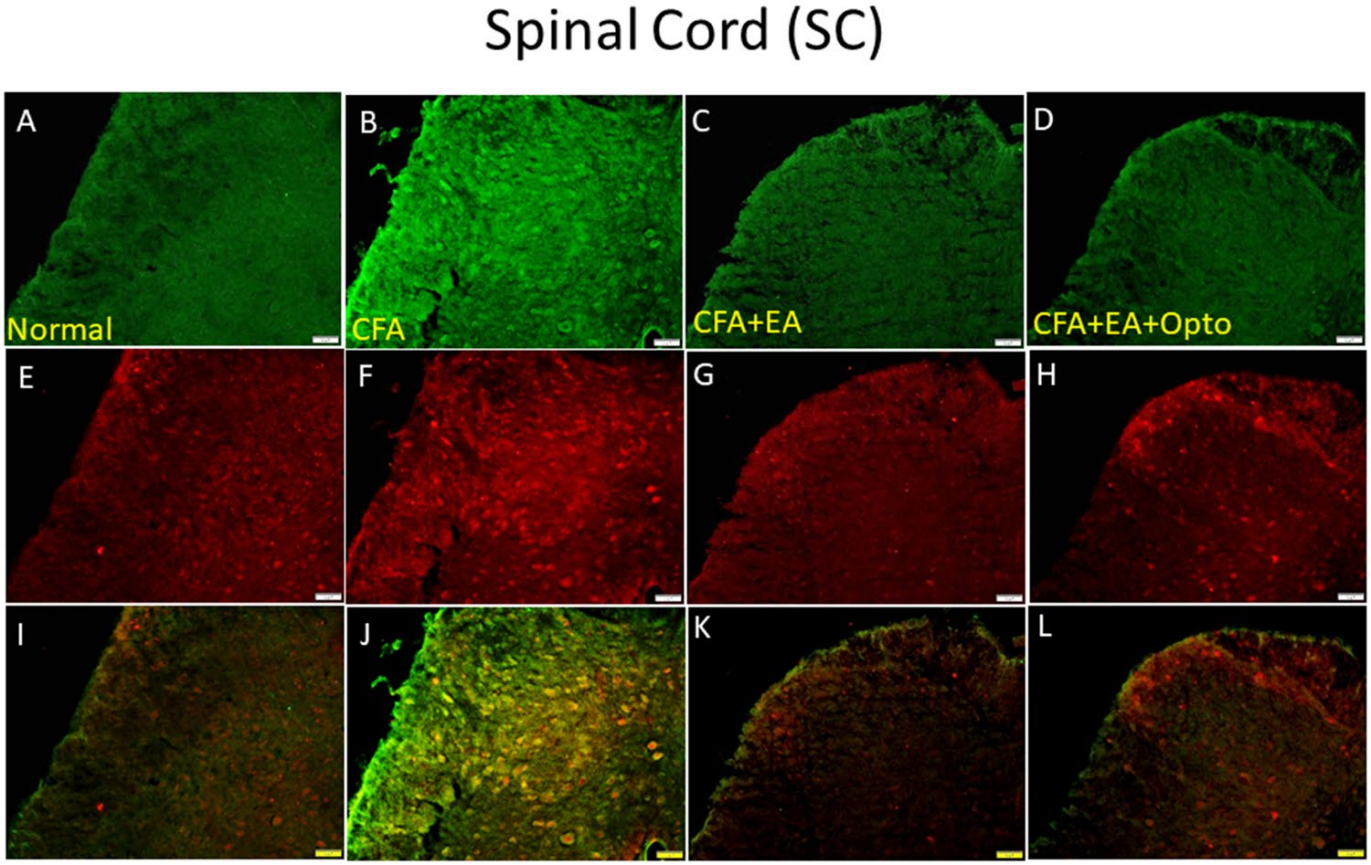
Immunofluorescence staining of pCaMKIIα, pERK, and double staining protein expression in the mice SC. (**A**–**D**) pCaMKIIα, (**E**–**H**) pERK, and (**I**–**L**) pCaMKIIα/pERK double staining, immuno-positive (green, red or yellow) signals in the mice SC region. Scale bar means 100 μm. n = 4 in all groups.

We further conducted immunofluorescence staining to examine the patterns of these expression changes in SC. Consistent with Western blotting results, pCaMKIIα immunoexpression in the SC (green fluorescence, Fig. 5B, n = 3) was elevated by CFA compared to control mice (green fluorescence, Fig. 5A, n = 3), and this increase was reversed by EA and optogenetic stimulation (Fig. 5C,D, respectively, n = 3 mice per group). These data support the involvement of SC neuronal activity in both the progression of CFA-induced inflammatory pain and the therapeutic response to AE. The expression level of pERK1/2 exhibited a qualitatively similar response to CFA, AE, and optogenetic stimulation (Fig. 5E–H, red fluorescence, n = 3). Moreover, merging of fluorescent images indicated co-expression of pCaMKIIα and pERK in SC neurons following CFA injection and simultaneous downregulation by EA and optogenetic stimulation (Fig. 5I–L, yellow signals in merged images, n = 3).

### EA and optogenetic stimulation had reciprocal effects on pain-associated signaling pathways in the SSC

**Figure 6:**
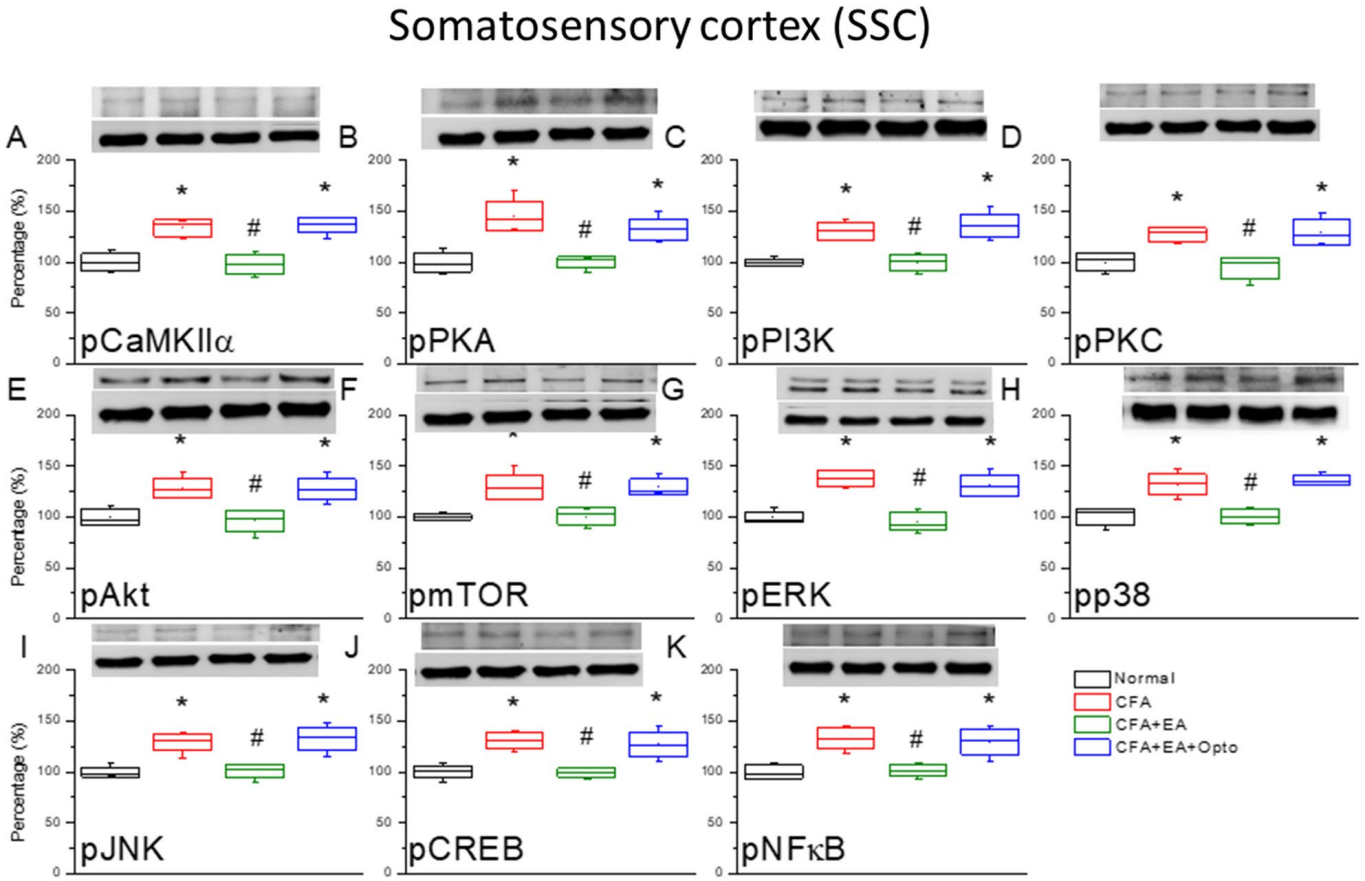
The levels of pCaMKIIα and related molecules in the mice SSC. The western blot bands have four lanes of protein expression in order of Normal, CFA, CFA + EA, and CFA + EA + Opto groups. There are significant increases of (**A**) pCaMKIIα, (**B**) pPKA, (**C**) pPI3K, (**D**) pPKC, (**E**) pAkt, (**F**) pmTOR, (**G**) pERK, (**H**) pp38, (**I**) pJNK, (**J**) pCREB, and (**K**), pNFκB protein levels in CFA and CFA + EA + Opto groups. *Indicates statistical significance when compared with the normal group. ^#^Indicates statistical significance when compared to the CFA group. n = 6 in all groups. The full Western blot bands are supplied at the end of the supplementary file.

On day three after CFA injection, we dissected SSC sections, and estimated protein expression levels by Western blotting. Similar to response in lower brain regions, CFA significantly upregulated the expression levels of pCaMKIIα, pPKA, pPI3K, and pPKC (Fig. 6A–D, red columns, *p < 0.05 vs. controls, n = 6 mice per group), while EA reversed these changes (Fig. 6A–D, green column, ^#^p < 0.05 vs. CFA alone). Optogenetic activation of SSC after EA treatment also significantly enhanced the expression levels of all four molecules compared to the EA group (Fig. 6A–D, blue column, *p < 0.05). Injection of CFA also upregulated pAkt and pmTOR in the SSC (Fig. 6E,F, red column, *p < 0.05 vs. controls), while EA reversed these effects (Fig. 6E,F, green columns, ^#^p < 0.05 vs. CFA group) and re-activation of SSC neural circuits by optogenetic stimulation significantly reversed the attenuation induced by EA (Fig. 6E,F, blue column, *p < 0.05 vs. EA). Similarly, CFA upregulated pERK, pp38, and pJNK expression levels (Fig. 6G–I, blue columns, *p < 0.05 vs. control), while EA reversed these effects of CFA (Fig. 6G–I, green columns, ^#^p < 0.05) and optogenetic stimulation reversed the attenuation induced by EA (Fig. 6G–I, blue columns, *p < 0.05 vs. EA). The nuclear expression levels of pCREB and pNFκB in the SSC exhibited qualitative similar response patterns (Fig. 6J,K).

**Figure 7:**
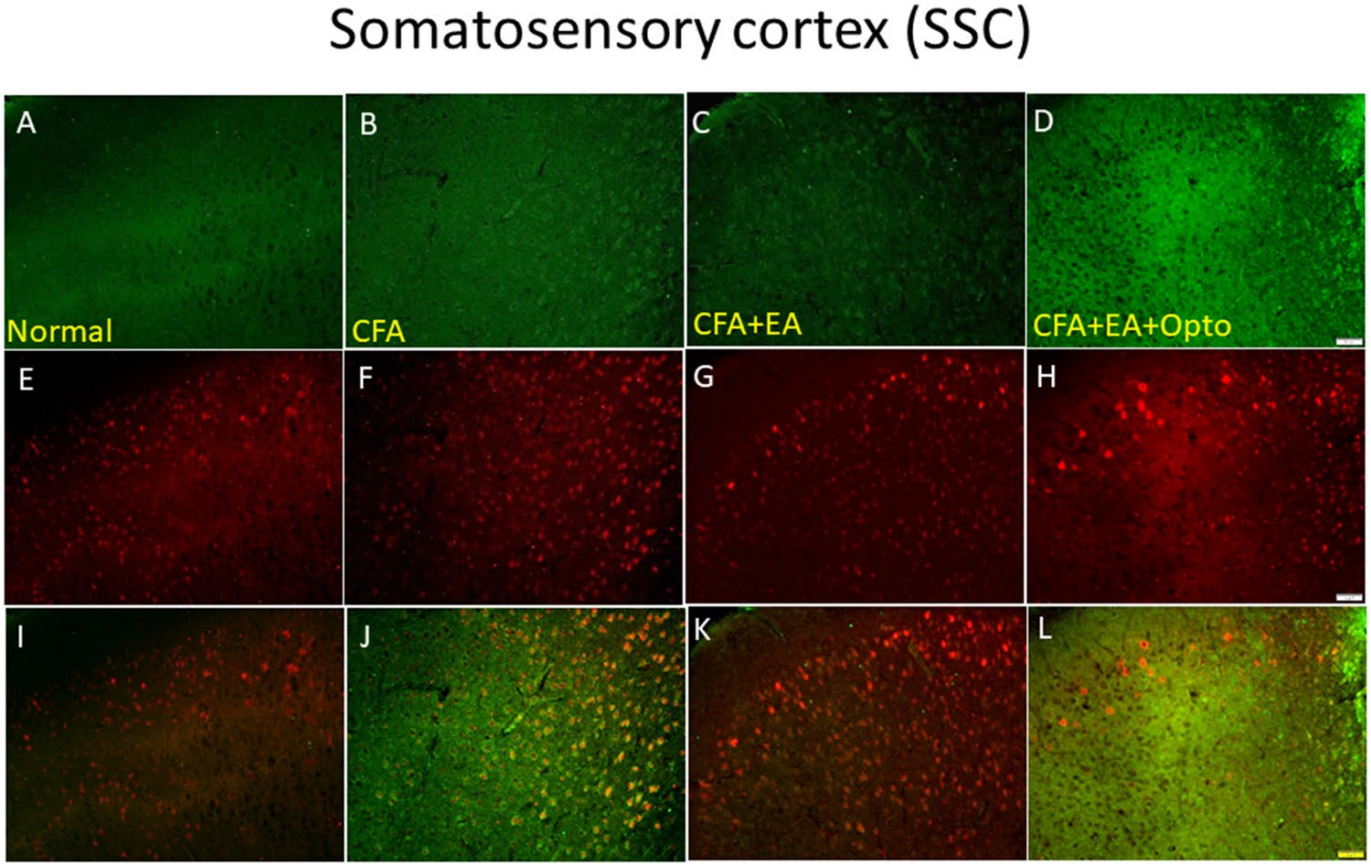
Immunofluorescence staining of pCaMKIIα, pERK, and double staining protein expression in the mice SSC. (**A**–**D**) pCaMKIIα, (**E**–**H**) pERK, and (**I**–**L**) pCaMKIIα/pERK double staining, immuno-positive (green, red or yellow) signals in the mice SSC region. Scale bar means 100 μm. n = 4 in all groups.

Next, we examined if higher expression of pCaMKIIα and pERK in the SSC following CFA injection was associated with inflammatory pain by measuring immunoexpression specifically in L2/3. Indeed, the ratio of pCaMKIIα-positive SSC neurons was significantly higher in the CFA group than the control group (Fig. 7A, green fluorescence) on day three after CFA administration (Fig. 7B, p < 0.05, n = 3 mice per group) and this effect was attenuated by EA, suggesting that EA analgesia is mediated in part by suppression of pCaMKIIα signaling in the SSC (Fig. 7C). Moreover, pCaMKIIα expression in L2/3 was enhanced by optogenetic stimulation following EA (Fig. 7D) concomitant with reversal of EA analgesia, providing further evidence that CFA-induced inflammatory pain is mediated in part by enhanced pCaMKIIα signaling in the SSC. Similar to pCaMKIIα, the number of pERK-positive neurons (Fig. 7E, red fluorescence, n = 3 mice per group) was dramatically increased in CFA-injected mice compared to controls (Fig. 7F) and this effect was attenuated by EA (Fig. 7G). Also in accord with changes in pCaMKIIα, optogenetic stimulation reversed the effect of EA on pERK (Fig. 7H). Qualitatively similar response patterns were observed in merged images of dual-strained sections of SSC (Fig. 7I–L, yellow signals).

### EA and optogenetic stimulation modulated pain-associated signaling pathways in the ACC

**Figure 8:**
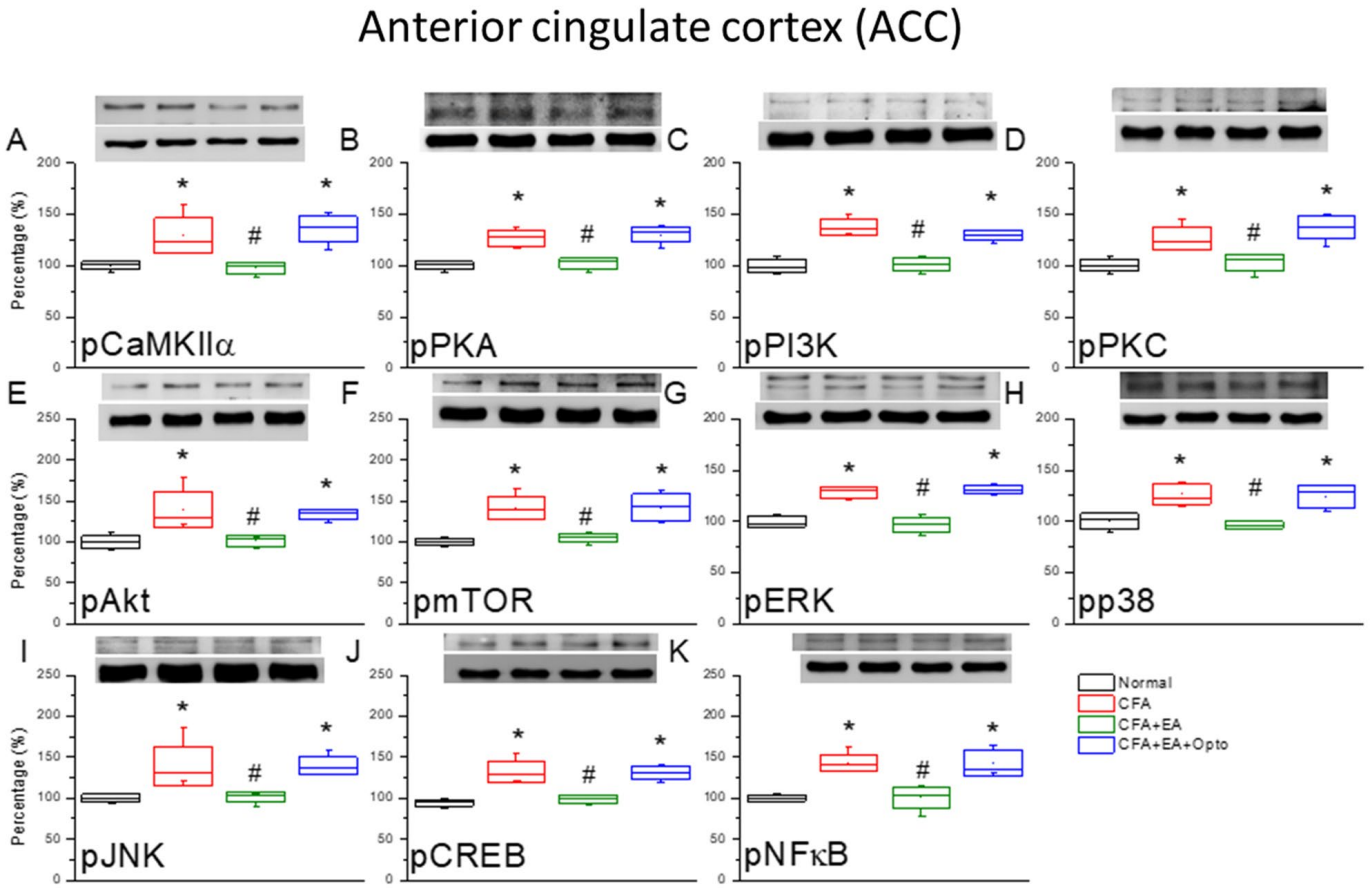
The levels of pCaMKIIα and related molecules in the mice ACC. The western blot bands have four lanes of protein expression in order of Normal, CFA, CFA + EA, and CFA + EA + Opto groups. There are significant increases of (**A**) pCaMKIIα, (**B**) pPKA, (**C**) pPI3K, (**D**) pPKC, (**E**) pAkt, (**F**) pmTOR, (**G**) pERK, (**H**) pp38, (**I**) pJNK, (**J**) pCREB, and (**K**), pNFκB protein levels in CFA and CFA + EA + Opto groups. *Indicates statistical significance when compared with the normal group. ^#^Indicates statistical significance when compared to the CFA group. n = 6 in all groups. The full Western blot bands are supplied at the end of the supplementary file.

The ACC functions in the sensory and emotional processing of pain. Therefore, we examined these same signaling pathways in the ACC of control, CFA, EA, and optogenetic group mice (Fig. 8, n = 6 mice per group). As in the SSC, the ACC region of CFA group mice expressed significantly higher pCaMKIIα levels (Fig. 8A, red column, *p < 0.05) than controls (Fig. 8A, black column), and this upregulation was reversed by EA (Fig. 8A, green column, ^#^p < 0.05 vs. CFA alone), in accord with the effects on pain-related behaviors. Optogenetic activation of the SSC also reversed the effect of EA on pCaMKIIα expression (Fig. 8A, red column, *p < 0.05 vs. EA). The expression levels of pPKA, pPI3K, and pPKC exhibited qualitatively similar response patterns (Fig. 8B–D). Furthermore, pmTOR and pPI3K expression levels were also enhanced by CFA injection compared to controls (Fig. 8E,F, red columns, *p < 0.05). Again, EA reversed these effects of CFA (Fig. 8E,F, green columns, ^#^p < 0.05), while subsequent optogenetic activation of the SSC reversed the suppressive effects of EA (Fig. 8E,F, blue columns, *p < 0.05). Expression levels of pERK, pp38, and pJNK were also increased by CFA (Fig. 8G–I, red columns, *p < 0.05 vs. control), and these effects were reversed by EA (Fig. 8G–I, green columns). However, optogenetic stimulation had no further influence on expression (blue columns in Fig. 8G–I). Qualitatively similar responses were observed for the transcription factors pCREB and pNFκB (Fig. 8J,K).

**Figure 9:**
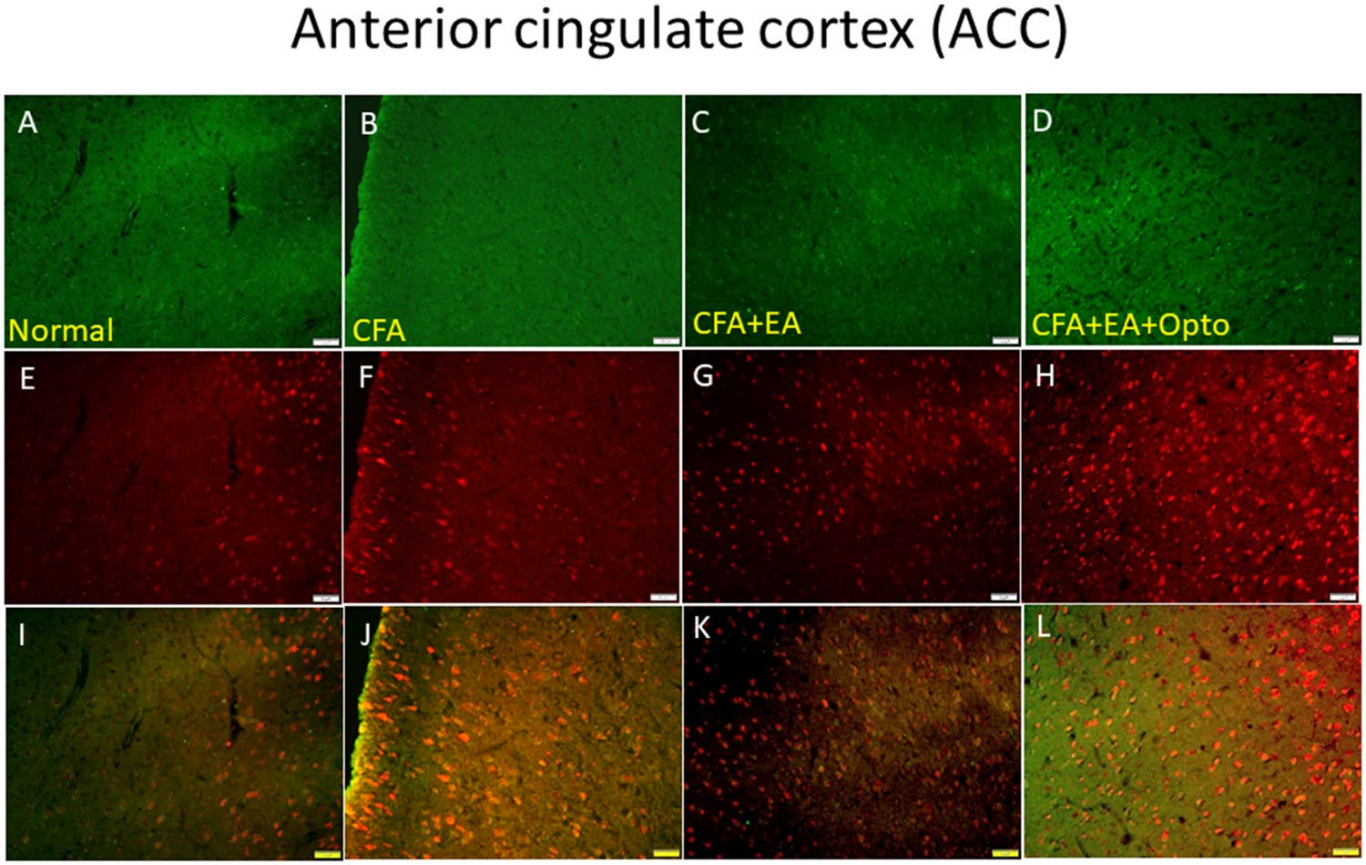
Immunofluorescence staining of pCaMKIIα, pERK, and double staining protein expression in the mice ACC. (**A**–**D**) pCaMKIIα, (**E**–**H**) pERK, and (**I**–**L**) pCaMKIIα/pERK double staining, immuno-positive (green, red or yellow) signals in the mice ACC region. Scale bar means 100 μm. n = 4 in all groups.

Finally, we conducted immunostaining of the ACC to confirm Western blotting results and elucidate the cellular distributions of these CFA-regulated signaling factors. Immunoexpression of pCaMKIIα in ACC sections was significantly enhanced by CFA injection (Fig. 9B, green fluorescence, n = 3 mice per group) compared to control mice (Fig. 9A). This elevated immunoexpression of pCaMKIIα in the ACC was attenuated by EA (Fig. 9C), while optogenetic stimulation had no additional effect (Fig. 9D). Similarly, pERK expression (Fig. 9E, normal group, red fluorescence, n = 3 mice per group) was substantially upregulated by CFA injection (Fig. 9F). Like pCaMKIIα expression, this elevated pERK expression in the ACC was reversed by EA (Fig. 9G) while optogenetic stimulation had no effect (Fig. 9H). Qualitatively similar response patterns were observed in dual-stained sections (Fig. 9I–L, yellow signals in merged images).

## Discussion

In this study, we confirmed that SSC neurons played a vital role in the central sensitization of the CFA-induced mice inflammatory pain model, as we showed increased mechanical and thermal hyperalgesia in these mice. Results showed that EA treatment significantly attenuated mechanical and thermal hyperalgesia. The direct activation of SSC neurons using the optogenetics activation methodology of the CaMKII signaling pathway was also repeatedly enough to reverse EA analgesia. Moreover, the CFA injection significantly increased circulating IL-6 and TNF-α. Although EA treatment reliably attenuated the increase in inflammatory mediators, optogenetic activation did not change these phenomena. We also confirmed that the pCaMKII signaling pathway and associated molecules were increased in inflamed mice DRG, SC, SSC, and ACC. Therefore, EA reliably abolished the increase in CaMKII and related molecules. Furthermore, the optogenetic activation of SSC reversed the phenomena in SSC and ACC, suggesting a higher cortical modulation. Hence, our information indicates that targeting SSC neurons reverses the analgesic effect of EA in mice inflammatory pain model.

Optogenetics has been created as a novel technique to investigate specialized cellular activities or circuits^24^. It is a biological method to regulate the activity of cells using different light wavelengths. Opsins, which are light-sensitive molecules, have also been used in target cells to specifically regulate biochemical signaling pathways. Besides, a recent article has indicated that the optogenetic activation of contralateral prelimbic cortex excitatory neurons has analgesic effects on chronic pain mice^25^. In the previously understudied spinal cord-injured rats, a higher spontaneous firing rate in SSC neurons and mechanical hyperalgesia was observed^26^. Precise optogenetic stimulation of the dorsal medial PFC also had antinociceptive effects in chronic pain mice^27^. Likewise, although Okada et al., indicated an increase in neuronal activity and connectivity in SSC during pain induction, chemogenetic manipulation significantly reduced pain thresholds^28^. Jin et al. observed an increased activity of the SSC excitatory signals to the striatum in the understudied CFA inflammatory pain model as well^29^. In this study, our data indicated that optogenetic activation in layer 2/3 of SSC significantly reversed EA analgesia. Another recent article reported that the optogenetic activation of ACC inhibitory neurons had an antinociceptive effect on chronic mice pain. Moreover, an up-regulation in ACC neurons was observed during chronic pain conditions^30–32^. Similarly, Elina et al., reported optogenetic inhibition of the ACC-attenuated mechanical and thermal hyperalgesia in nerve injury-induced neuropathic pain models^33^. In this study, we indicated that the optogenetic activation of SSC further increased the neural circuit connectivity to ACC, thereby reversing EA analgesia.

The noticeable discovery during our study was that local CFA injections initiated hyperalgesia and circulating inflammation, which induced central sensitization in the mice brain. Inflammation also induced mechanical and thermal hyperalgesia accompanied by increased IL-6 and TNF-α in the mice plasma, suggesting EA as an anti-inflammatory technique. Recently articles reported that local inflammation in mice increased circulating IL‑6 and TNF-α levels^34^. Additionally, although the administration of IL-6 induced mechanical and thermal hyperalgesia, IL-6 blockage produced analgesic effects^35^. As previously reported, the injection of IL-6 antibody reliably attenuated peripheral nerve injury-induced mechanical hyperalgesia^36^. Blocking TNF-α also abolished neuropathic pains through a decrease in pp38 and JNK signaling pathways in rat DRG^37^. Furthermore, Ko et al., indicated that TNF-α and its receptors were highly associated with pain, following hip fracture^38^. Our previous publications showed that IL‑6 and TNF-α were augmented during mice cold stress and inflammatory pain models. These inflammatory mediators were reported to be further attenuated through EA treatments as well^15^.

Our previous publication suggested that the transient receptor potential cation channel subfamily V member 1 (TRPV1), a Ca^2+^ permeable ion channel, including related molecules, was increased in the inflamed mice DRG and SC. Thus, TRPV1 and associated molecules were reported as inflammatory biomarkers in both peripheral sites and the brain^19,39^. In this study, we showed that pCaMKIIα and related nociceptive molecules were all increased in the CFA-induced inflammatory pain model. EA significantly alleviated these molecules augmentation. Besides, the optogenetic activation of SSC did not alter the peripheral reduction of these aforementioned molecules through EA. Data also indicated that EA can reduce inflammatory pain through a downregulation of the pCaMKIIα signaling pathway at peripheral DRG and central SC levels. Furthermore, at the higher pain matrix, CFA injections increased the pCaMKIIα, including associated molecules in mice SSC and ACC. Although EA significantly reduced the augmentation, further reversal was observed after optogenetic activation. These results propose that after EA treatments, the advanced activation of SSC can again be initiated through the pCaMKIIα signaling pathway in both SSC and ACC. Data also implied that activation of SSC can activate ACC neural circuits. In agreement, Xiao et al. reported coding roles between SSC and ACC during cortical pain processing^31^.

**Figure 10:**
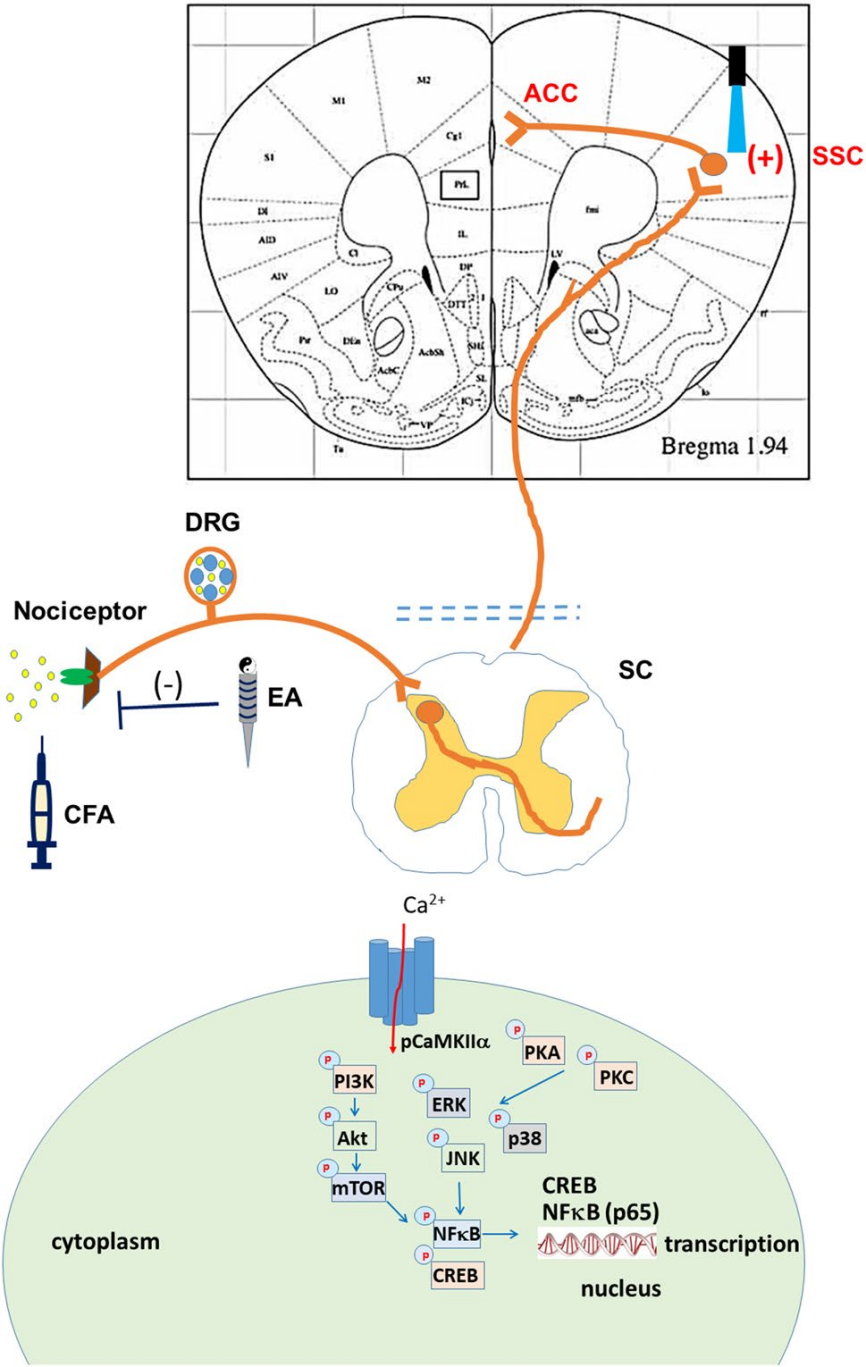
pCaMKIIα and related molecular pathways in mice.

In summary, our results have revealed analgesic and anti-inflammatory effects of EA using mice inflammatory pain model. Although optogenetic activation in higher cortical SSC reversed the analgesic effect of EA, inflammation persisted. The augmentation of pCaMKIIα and associated nociceptive molecules were also increased in DRG, SC, SSC, and ACC. Additionally, although these molecules were further attenuated through EA treatment, showing a similar tendency with pain behavior, optogenetic activation of SSC reversed this EA effect on SSC and ACC but not on DRG and SC. These conclusions therefore recommend significant circuit mechanisms that induce inflammatory pain and EA analgesia (Fig. 10).

## Methods

### Animals

There are total 36 female C57BL/6 mice, aged 8–12 weeks, were used in the current study. After arriving, the mice were kept in a 12 h light–dark cycle with food and water ad libitum. A sample size of nine animals per group was calculated as the number required for an alpha of 0.05 and a power of 80%. In addition, the number of animals used here and their suffering were minimized. The laboratory workers were blind to treatment allocation during the experiments and analysis. The use of these animals was approved by the Institute of Animal Care and Use Committee of China Medical University (Permit no. CMUIACUC-2019-106), Taiwan, following the Guide for the use of Laboratory Animals (National Academy Press). This study is reported in accordance with ARRIVE guidelines. Mice were subdivided into four groups: Normal mice injected with normal saline (Group 1: Normal); CFA-induced inflammatory pain group (Group 2: CFA); CFA-induced inflammatory pain treated with EA group (Group 3: CFA + EA), and EA mice treated with optogenetic group (Group 4: CFA + EA + Opto).

### Viral infection

Mice were anesthetized using isoflurane, after which their heads were fixed in a stereotaxic cannula, and the cannula was implanted at the SSC site. The stereotaxic cannula was subsequently put 0.5 mm posteriorly and 1.5 mm laterally from the bregma at a depth of 175 μm below the cortical surface. This cannula was a 23 gage, comprising a 2 mm stainless steel. Then, it was fixed at the skull using a dental cement. Afterward, the cannula injection was inserted and connected to a Hamilton syringe with a PE tube (PE10, Portex). Entirely, 0.3 μl of the viral solution was injected over a period of 3 min using a syringe pump (KD Scientific). After the injection, the cannula was left at the SSC for an additional 2 min to allow the drug to diffuse. Weeks later, mice brains containing SSC were harvested to verify the injection site. The virus injection at the SSC; AAV, was obtained from the UNC Vector Core (University of North Carolina School of Medicine). Next, 0.3 μl AAV5-CaMKII-hChR2(H134R)-eYFP-EPRE and AAV5-CaMKII-eYFP were injected into SSC for four weeks. We followed a previously reported method for this analysis, blue light optogenetic stimulation was performed in the SSC after EA treatment in 20 min for 3 consecutive days^6^. Figure 1A and B illustrate the experimental protocol.

### Inflammatory pain model and Bio-Plex ELISA

Mice were injected either with 20 μl saline (pH 7.4, buffered with 20 mM HEPES) or CFA 20 μl (complete Freund’s adjuvant; 0.5 mg/ml heat-killed M. tuberculosis (Sigma, St. Louis, MO) suspended in oil: saline 1:1 emulsion) in the plantar surface of the hind paw. CFA was used to induce mice inflammatory pain model. After induction of inflammatory pain at day 3, mice plasma was collected and analyzed on Q-view cytokine assays (Q-view, CA, USA).

### EA treatments

The mice were anaesthetized with 5% isoflurane for induction, and then maintained in 1% isoflurane. Under anesthesia, a pair of stainless steel acupuncture needles (1″ inch, 36G, YU KUANG, Taiwan) were bilaterally inserted at a depth of 3–4 mm into the murine equivalent of the human ST36 acupoints. Murine ST36 acupoint is located approximately 3–4 mm below and 1–2 mm lateral to the midpoint of the knee. In the EA group, electrical stimuli were delivered by Trio 300 stimulator (Ito, Japan) at an intensity of 1 mA for 20 min at 2 Hz with a pulse width of 100 μs. The EA treatment caused slight visible muscle twitching around the area of insertion. The EA stimulation was applied thrice from day 1 to 3, following the CFA injection.

### Pain behavior test

Mice mechanical and thermal pain behaviors were examined 4 times from day 0 to 3 throughout the experiment before and after the induction of the inflammatory pain model. All mice were moved to the behavior analysis room, and were improved to the situation for at least 30 min before behavior tests. All tests were done at room temperature and the stimuli were applied only when the mice were calm but not sleeping or grooming. First, the von Frey filament test was done. Mechanical sensitivity was measured by trying the force of responses to stimulus with 3 applications of the electronic von Frey filament (IITC Life Science Inc., USA). Mice were located onto a steel mesh (75 × 25 × 45 cm) and covered with a plexiglass cage (10 × 6 × 11 cm). Mice were then mechanically stimulated by the tip of the filament at the plantar region of the right hind paw. The filament gram counts were documented when the stimulation caused the subject to withdraw its hind paw. Second, the Hargreaves’ test was used to quantity thermal pain behavior by testing the time of response to thermal stimulation with 3 applications using Hargreaves' test IITC analgesiometer (IITC Life Sciences, SERIES8, Model 390G). The mice were placed in a plexiglass cage on top of a glass sheet. The thermal stimulator was situated under the glass sheet and the focus of the projection bulb was aimed exactly at the middle of the plantar surface of the right hind paw. A cut-off time of 20 s was set to avoid tissue injury. In the thermal paw withdrawal test, the nociceptive threshold was measured using the latency of paw withdrawal upon stimulus, and was recorded when the continuous applied heat stimulus caused the subject to withdraw its hindpaw.

### Western blot analysis

The mice were anaesthetized with 1% isoflurane and cervical dislocation. The L3-L5 DRG, L3-L5 SC, total SSC, and total ACC tissues were directly excised to extract proteins. Tissues were initially placed on ice and later stored at − 80 °C, pending protein extraction. Total proteins were homogenized in cold radioimmunoprecipitation (RIPA) lysis buffer containing 50 mM Tris–HCl pH 7.4, 250 mM NaCl, 1% NP-40, 5 mM EDTA, 50 mM NaF, 1 mM Na_3_VO_4_, 0.02% NaN_3_, and 1 × protease inhibitor cocktail (AMRESCO). The extracted proteins were subjected to 8% SDS-Tris glycine gel electrophoresis and transferred to a PVDF membrane. The membrane was blocked with 5% non-fat milk in TBS-T buffer (10 mM Tris pH 7.5, 100 mM NaCl, 0.1% Tween 20), incubated with a primary antibody in TBS-T with 1% bovine serum albumin (BSA) for 1 h at room temperature antibody against pCaMKIIα (∼ 55 kDa, 1: 1000, Alomone, Israel), pPKA (∼ 40 kDa, 1: 1000, Alomone, Israel), pPKC (∼ 100 kDa, 1: 1000, Millipore, USA), pPI3K (∼ 125 kDa, 1: 1000, Millipore, USA), pERK1/2 (∼ 42–44 kDa, 1: 1000, Millipore, USA), pp38 (∼ 41 kDa, 1: 1000, Millipore, USA), pJNK (∼ 42 kDa, 1: 1000, Millipore, USA), pAkt (∼ 60 kDa, 1: 1000, Millipore, USA), pmTOR (∼ 60 kDa, 1: 500, Millipore, USA), and pNFκB (∼ 65 kDa, 1: 1000, Millipore, USA), in TBS-T with 1% bovine serum albumin. The Western blots membranes were cut prior to hybridization with antibodies. Peroxidase-conjugated anti-rabbit antibody, anti-mouse antibody or anti-goat antibody (1: 5000) was used as the appropriate secondary antibody. The bands were visualized by an enhanced chemiluminescent substrate kit (PIERCE) with LAS-3000 Fujifilm (Fuji Photo Film Co., Ltd.). Where applicable, the image intensities of specific bands were quantified with NIH ImageJ software (Bethesda, MD, USA). β-actin or α-tubulin was served as internal control.

### Immunofluorescence

Mice were euthanized with a 5% isoflurane via inhalation and intracardially perfused with normal saline followed by 4% paraformaldehyde. The brain was immediately dissected and post fixed with 4% paraformaldehyde at 4 °C for 3 days. The tissues were placed in 30% sucrose for cryoprotection overnight at 4 °C. The brain was embedded in an Optimal cutting temperature (OCT) compound and rapidly frozen using liquid nitrogen before storing the tissues at − 80 °C. Frozen segments were cut at 20-μm width on a cryostat then instantaneously placed on glass slides. The samples were fixed with 4% paraformaldehyde, then incubated with a blocking solution, consisting of 3% BSA, 0.1% Triton X-100, and 0.02% sodium azide, for 1 h at room temperature. After blocking, the samples were incubated with the primary antibody (1:200, Alomone), pCaMKIIα and pERK, prepared in 1% bovine serum albumin solution at 4 °C overnight. The samples were then incubated with the secondary antibody (1:500), 488-conjugated AffiniPure donkey anti-rabbit IgG (H + L), 594-conjugated AffiniPure donkey anti-goat IgG (H + L) and Peroxidase-conjugated AffiniPure donkey anti-mouse IgG (H + L) for 2 h at room temperature before being fixed with cover slips for immunofluorescence visualization. The samples were observed by an epi-fluorescent microscope (Olympus, BX-51, Japan) with 20 × numerical aperture (NA = 1.4) objective. The images were analyzed by NIH Image J software (Bethesda, MD, USA).

### Statistical analysis

Statistical analysis was performed using the SPSS statistic program. All statistic data are presented as the mean ± standard error (SEM). Shapiro–Wilk test was performed to test the normality of data. Statistical significance among all groups was tested using the repeated measure ANOVA test, followed by a post hoc Tukey’s test. Values of p < 0.05 were considered statistically significant (Supplementary figures).

## Supplementary Information

Supplementary Figures.

## Data Availability

The datasets supporting the conclusions of this article are included within the article.
