# Supplementary figures and images for: Optogenetic modulation of electroacupuncture analgesia in a mouse inflammatory pain model
Source: Sci Rep. 2022 May 31;12:9067. doi: 10.1038/s41598-022-12771-8 (PMC9156770; doi:10.1038/s41598-022-12771-8)

# Figure 2: DRG

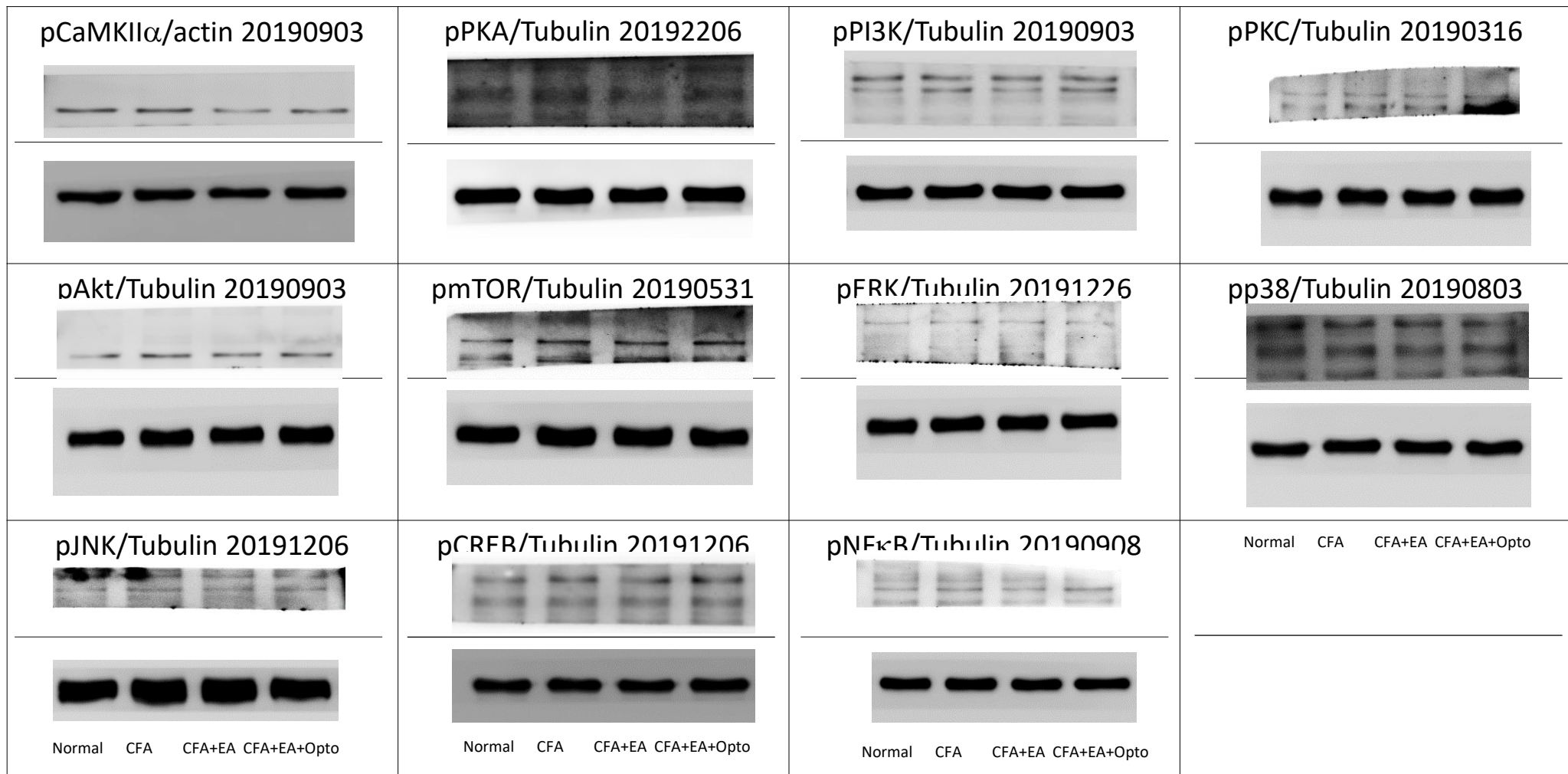

# Figure 4: SC

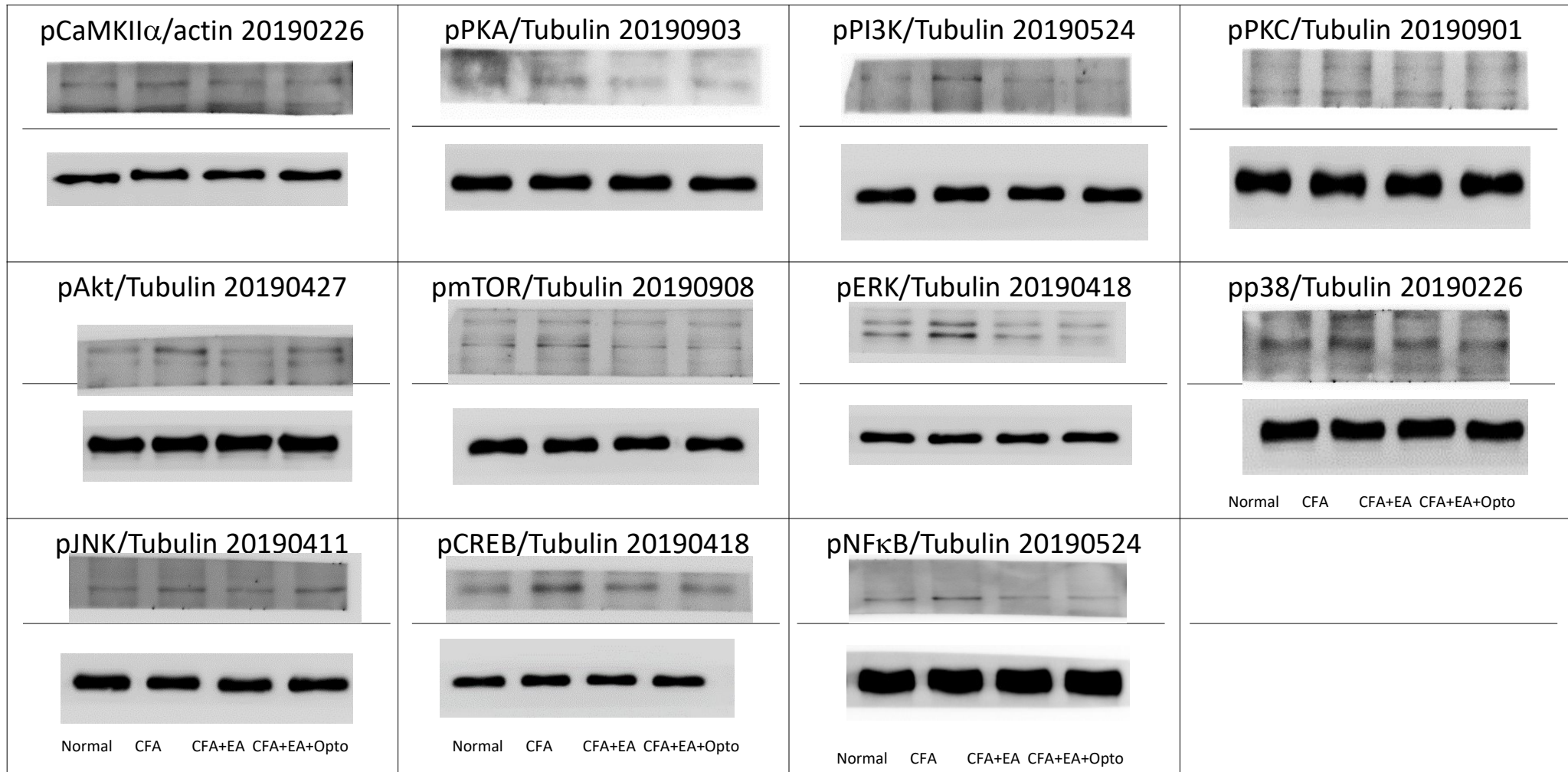

# Figure 6: SSC

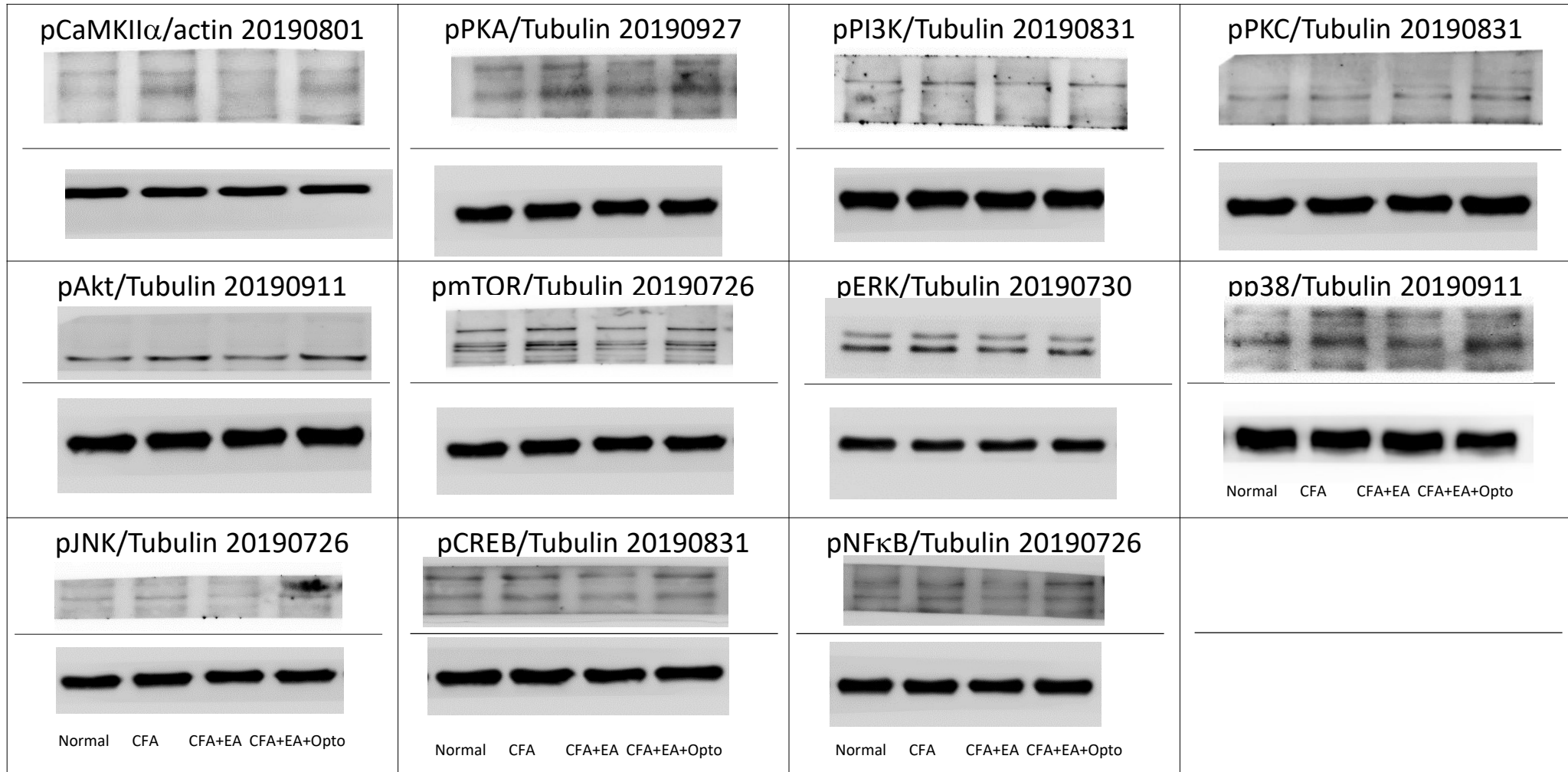

# Figure 8: ACC

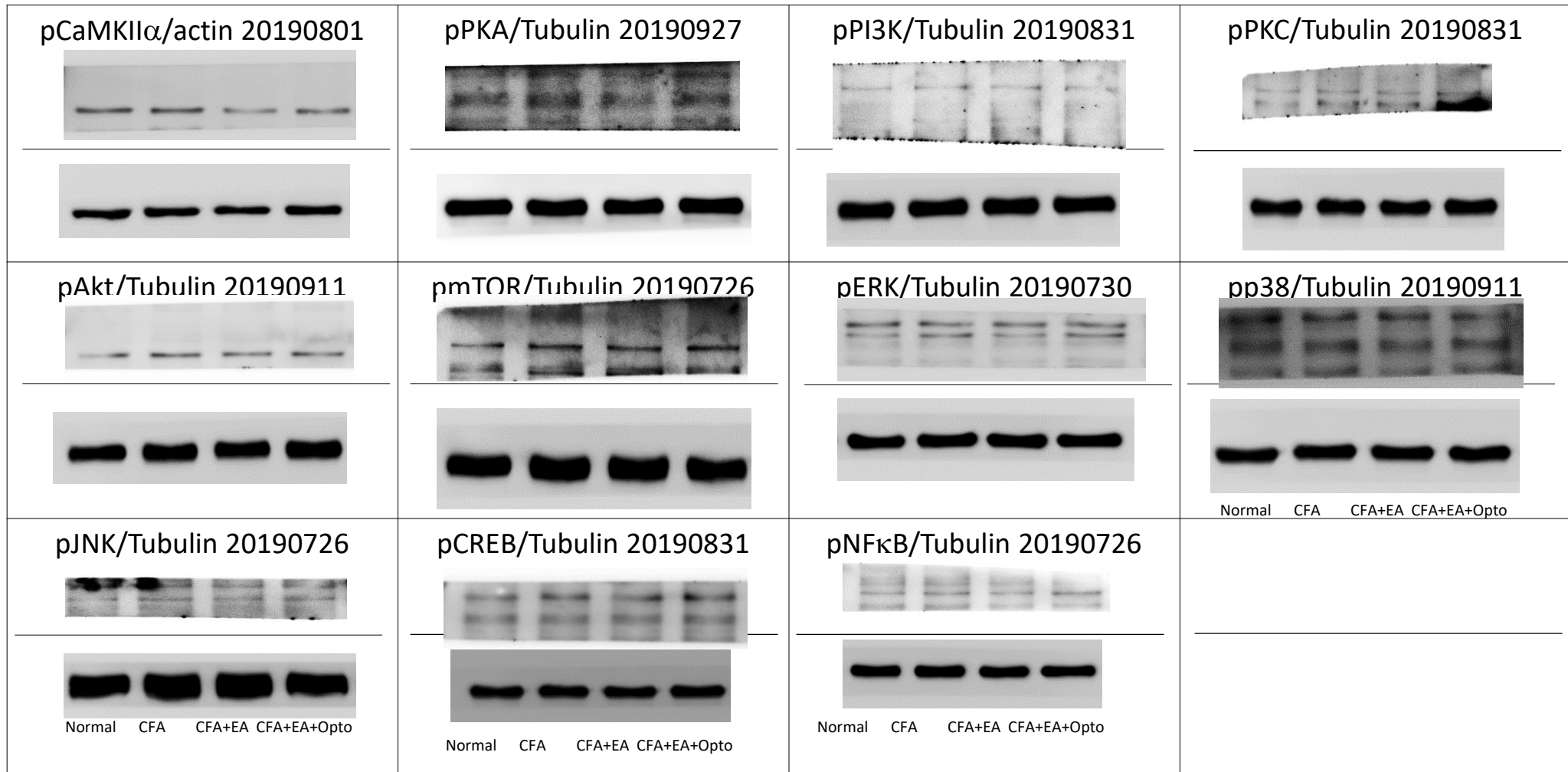

Supplement: Supplementary file 1 — Supplementary Figures. [file 41598_2022_12771_MOESM1_ESM.pdf]
